# Imaging Ischemic and Hemorrhagic Disease of the Brain in Dogs

**DOI:** 10.3389/fvets.2020.00279

**Published:** 2020-05-27

**Authors:** Susan A. Arnold, Simon R. Platt, Karine P. Gendron, Franklin D. West

**Affiliations:** ^1^Department of Veterinary Clinical Sciences, University of Minnesota Twin Cities, St. Paul, MN, United States; ^2^Department of Small Animal Medicine and Surgery, University of Georgia, Athens, GA, United States

**Keywords:** ischemic stroke, hemorrhagic stroke, canine, MRI, CT

## Abstract

Strokes, both ischemic and hemorrhagic, are the most common underlying cause of acute, non-progressive encephalopathy in dogs. In effect, substantial information detailing the underlying causes and predisposing factors, affected vessels, imaging features, and outcomes based on location and extent of injury is available. The features of canine strokes on both computed tomography (CT) and magnetic resonance imaging (MRI) have been described in numerous studies. This summary article serves as a compilation of these various descriptions. Drawing from the established and emerging stroke evaluation sequences used in the investigation of strokes in humans, this summary describes all theoretically available sequences. Particular detail is given to logistics of image acquisition, description of imaging findings, and each sequence's advantages and disadvantages. As the imaging features of both forms of strokes are highly representative of the underlying pathophysiologic stages in the hours to months following stroke onset, the descriptions of strokes at various stages are also discussed. It is unlikely that canine strokes can be diagnosed within the same rapid time frame as human strokes, and therefore the opportunity for thrombolytic intervention in ischemic strokes is unattainable. However, a thorough understanding of the appearance of strokes at various stages can aid the clinician when presented with a patient that has developed a stroke in the days or weeks prior to evaluation. Additionally, investigation into new imaging techniques may increase the sensitivity and specificity of stroke diagnosis, as well as provide new ways to monitor strokes over time.

## Introduction

Strokes, also commonly referred to as cerebrovascular accidents, are becoming increasingly recognized as a common cause of acute neurologic dysfunction in dogs. Frequency data are lacking, however, the incidence reported at one referral hospital was an estimated 1.5–2% of neurological referrals (1). Stroke is the sudden onset of focal neurological deficits resulting from an intracranial vascular event with clinical signs lasting for at least 24 h (1–3). Cerebrovascular disease refers to any abnormality in the brain resulting from a pathological process of the cerebral blood vessels, such as thrombosis, embolism, or hemorrhage (1–3).

It is suspected that the prevalence of strokes is higher than previously considered, as advanced imaging modalities have become more widely used and validated. The purpose of this review is to describe the imaging sequences, both established and emerging, that are available for evaluation of both ischemic and hemorrhagic strokes in dogs. Firstly, a summary of cerebral vascular anatomy as it applies to the development of strokes in dogs is followed by a review of the pathophysiology associated with the development of ischemic and hemorrhagic strokes in dogs. Subsequently, through a review of the sequences used in both human and veterinary medicine, the applications, benefits, drawbacks, and special considerations of each sequence are described. When possible, direct examples of their application in veterinary medical patients are described.

### Anatomy Review (Figure 1)

As the focus of this review is to describe established and emerging imaging modalities for characterizing vascular accidents, knowledge of the vascular anatomy of the brain is essential. Five major paired arteries supply the dog brain, namely, the rostral, middle, and caudal cerebral arteries and the rostral and caudal cerebellar arteries (1, 4). All but the caudal cerebellar arteries branch from a ring at the base of the brain, called the cerebral arterial circle or the Circle of Willis, which is formed from the paired internal carotid arteries and the basilar artery (1, 4). The caudal cerebellar arteries branch from the basilar artery (4).

**Figure 1:**
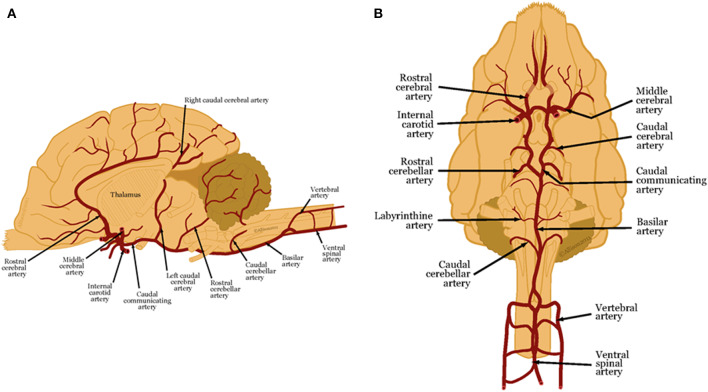
**(A)** Lateral and **(B)** ventral views of arterial blood supply to the canine brain. These illustrations were created by Allison L. Wright MS CMI, Athens, Georgia, USA and are reproduced with permission from the BSAVA Manual of Canine and Feline Neurology 4th edition.

These main arteries branch into deep and superficial perforating arteries. The proximal, distal and caudal deep perforating arteries arise from the caudal communicating arteries and the basilar artery and supply the thalamus, midbrain, and part of the pons (1, 4–6). Striate arteries emerge from the arterial circle, supplying the basal nuclei, internal capsule, amygdala, optic tract and thalamus (1, 4, 7). Superficial perforating arteries supply the brain surface and deep white matter (1, 6–8). The rostral cerebral artery sends branches that supply the rostral cerebral cortex, and deeper gray and white matter (4). The middle cerebral artery is the largest artery of the brain. As it courses along the ventral surface of the brain, it divides into two large branches that each supplies the whole cerebral cortex on the lateral surface of the cerebral hemisphere (4). The caudal cerebral artery supplies the medial aspect of the occipital lobe and caudal aspect of the marginal gyrus (4).

The rostral cerebellar artery arises either from the basilar artery or the caudal cerebral artery. The cerebellar arteries have variable distributions to the cerebellum and cerebellar peduncles. Generally, the rostral cerebellar artery supplies the cerebellar hemispheres and the vermis (9–13).

## Section 1: Stroke Classification Review

Broadly, two forms of stroke occur (ischemic and hemorrhagic), with major differences in frequency and imaging characteristics.

### Ischemic Strokes in Dogs: Epidemiology and Pathophysiology

The majority of strokes in dogs are ischemic strokes. Ischemic strokes result from vascular obstruction from emboli originating in other vascular beds, from the heart, or from local thrombus formation within a vessel (14, 15). Ischemic strokes are also referred to as cerebral ischemic strokes. As a result of ischemia, tissue infarction occurs. In dogs, one study found that the most common artery to develop emboli is the rostral cerebellar artery (47%), followed by the perforating arteries of the caudal thalamus and rostral brainstem (42%), the cerebral striate arteries (26%), middle cerebral artery (21%) and rostral cerebral artery (10%) (16). Depending on the location and extent of vessel occlusion, ischemic strokes are classified as territorial if affecting a large artery or lacunar if affecting a small, perforating artery (1).

Several underlying disorders in dogs are associated with embolic strokes, including septic diseases such as endocarditis, parasites such as *Dirofilaria immitis*, primary or metastatic neoplasia and various endocrine disorders and organ dysfunctions (9, 10, 13, 15, 17–23). Hypercoagulable states associated with hyperadrenocorticism, protein-losing nephropathy, hypothyroidism-induced and diabetes mellitus-induced atherosclerosis, as well as secondary hypertension have been detected in dogs with ischemic strokes, although the causal relationship between these conditions and ischemic stroke has not been definitively linked (13, 17, 21, 22, 24). In one retrospective study, a concurrent medical condition was found in 18/33 (54%) of dogs with ischemic strokes, with chronic kidney disease and hyperadrenocorticism being most common (13, 15).

In ischemic strokes, insufficient blood supply diminishes maintenance of normal cellular functions (1–3, 25–28). A reduction of cerebral blood flow below an ischemic threshold leads to hypoxia, decreased tissue glucose, and accumulation of potentially toxic metabolites that contribute to cell damage (1, 3). Hypoperfusion leads to anaerobic glycolysis and a decrease in production of adenosine triphosphate (ATP) (1, 3). Reduced intracellular ATP supply limits energy-dependent processes required to maintain homeostasis, including the maintenance of a resting transmembrane electrochemical potential, leading to cytotoxic edema (1). Focal ischemia also causes a breakdown of the blood-brain barrier that leads to a net influx of water into the affected tissue, resulting in vasogenic edema and extension of ischemic lesions (29). Complete obstruction of blood flow for >4–5 min produces irreversible cellular damage (1, 10).

Following arterial occlusion, a core of brain tissue dies rapidly as an area of infarction due to severe hypoperfusion (1, 3). Surrounding this core is an area of brain tissue that is hypoperfused but still viable, having retained borderline levels of blood flow and metabolic function (1, 26). This peripheral tissue is called the penumbra (1, 26). The penumbra is at risk for devitalization but is comprised of potentially salvageable tissue (1, 3, 16, 26). The evolution of the infarct core and penumbra is a dynamic process. The ratio between core and penumbra depends on the availability of collateral flow, and timing and extent of reperfusion of the ischemic tissue (30).

Differentiation of the infarct core and ischemic penumbra is based on the concept of cerebral vascular autoregulation (30–33). Complex neurobiochemical mechanisms maintain the stability of regional cerebral blood flow across a wide range of local metabolic activity and local arterial perfusion pressure (30–33). In the infarcted core, cerebral blood flow is low, leading to low cerebral blood volume and loss of ability to maintain autoregulatory vasodilatory compensation. Comparatively, in the penumbra, the autoregulation is intact or only mildly jeopardized (30–33).

Additionally, the relationship between cerebral blood flow, cerebral blood volume, and mean transit time differs between the infarcted core and the penumbra (34–38). Cerebral blood flow (CBF) is calculated as cerebral blood volume (CBV) divided by mean transit time (MTT) (34–38). MTT is the time difference between arterial inflow and venous outflow, designated by the average time required for a contrast bolus to cross a capillary network (30, 31, 34–39) It is the most sensitive measure used to evaluate cerebral blood flow abnormalities (34–38). The infarcted core has both decreased CBF and CBV due to loss of the autoregulatory ability (34–38). Decreased CBV is the most specific indicator that the area will infarct and therefore will be non-salvageable (34–38). Alternatively, the penumbra will have prolonged MTT, but the CBV is maintained or increased due to compensatory vasodilation (34, 40).

### Hemorrhagic Strokes in Dogs: Epidemiology and Pathophysiology

The prevalence of hemorrhagic strokes in dogs is unknown, but is estimated to account for the same percentage of strokes as it does in people (15–22%) (1, 9, 14, 41, 42). Hemorrhagic strokes are associated with a vessel rupture, with or without an obvious underlying cause, or a coagulopathy (1, 43–45) Hemorrhage can also occur as a secondary effect of ischemic strokes following reperfusion or if venous drainage is occluded (1).

There are numerous reported underlying etiologies for hemorrhagic strokes in dogs. Both primary and metastatic brain tumors can develop spontaneous intracranial hemorrhage (1, 46). Extracranial diseases that lead to disseminated intravascular coagulopathy or other causes of spontaneous bleeding, such as ingestion of anticoagulant rodenticide, can result in spontaneous intracranial hemorrhage. Additionally, bacterial infection, congenital vascular malformations, necrotizing vasculitis and brain atrophy leading to tearing of blood vessels have been reported as causes of hemorrhagic strokes (1, 45, 47, 48). In people, the main primary cause of hemorrhagic strokes is spontaneous rupture of an otherwise normal vessel secondary to hypertension. Conversely, hypertension leading to primary hemorrhagic strokes is rarely reported in dogs (1, 43, 46). Cerebral amyloid angiopathy results from amyloid deposits in cerebral arteries, leading to weakening of arterial walls (1). This condition is a common cause of intracranial hemorrhage in people and has been reported in a population of older dogs (1, 45, 49, 50). Hypertensive lesions are typically deep (such as in the thalamus and basal nuclei) and cerebral amyloid angiopathy spares these regions, being typically at the gray/white matter junction (51).

Hemorrhagic stroke results in extravasation of blood and formation of an intraparenchymal hematoma or diffuse infiltrate within the parenchyma (25, 45, 52). If the vessel rupture or coagulopathy occurs in the ventricles, subdural or subarachnoid space, then an extraparenchymal hematoma develops (25, 45, 52). Hemorrhagic strokes lead to an increase in cerebral volume, brain edema, and herniation (25, 45, 52). Clot expansion occurs mainly within the first 6 h of hemorrhage. It is often self-limiting due to increased cerebral perfusion pressure and brain tissue elastic resistance. Edema surrounding the clot can develop over several days, and ischemia can occur as a consequence of compressed brain tissue or limited blood flow (1, 49).

## Section 2: Established Stroke Imaging Modalities

According to established guidelines of the National Institute of Neurological Disorders and Stroke and the American Stroke Association, components of a hyperacute stroke imaging evaluation should include parenchymal, penumbral, susceptibility-weighted imaging and vascular imaging in a single study, consisting of either multimodal computed tomography (CT) or magnetic resonance (MR) (30, 53). These components fulfill the following criteria: (1) characterize the form of stroke (ischemic or hemorrhagic) and exclude other ischemic stroke mimics; (2) provide reliable information about the location and extent of ischemia in ischemic strokes; (3) identify the existence and extent of potentially salvageable brain; and (4) identify the site of vascular occlusion and degree of collateral flow (30, 53)

## Evaluating Parenchymal Changes

### Computed Tomography (Figure 2)

In people, non-contrast CT (NCT) is typically used to rule out hemorrhage and stroke mimics and to potentially detect the presence of early, subtle acute ischemic signs (30, 54–56). A major benefit of CT imaging is the rapidity of image acquisition; a complete protocol using NCT, perfusion CT (CTP), and CT-angiography (CTA) can be performed in <10 min (30, 53).

**Figure 2:**
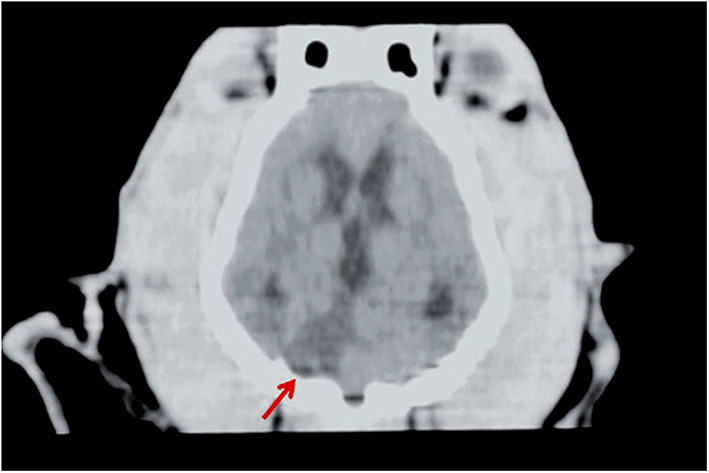
CT of a dog head, dorsal plane reconstruction. In the right lateral aspect of the cerebellum, there is a triangular, sharply margined and hypoattenuating lesion (red arrow) corresponding to an ischemic stroke in the territory of the right cerebellar artery. Image courtesy Wiley (Paul AEH, Lenard Z and Mansfield CS. Computed tomography diagnosis of eight dogs with brain infarction. Aust Vet J 2010; 88(10): 374-380.

NCT is used primarily based on its high sensitivity for detecting hemorrhage due to differences in Hounsfield units (HU) of acute hemorrhage (40–60 HU) compared to gray (39 HU) or white (32 HU) matter (57). Within hours of the onset of hemorrhage, attenuation of the hematoma rapidly increases up to 60–80 HU due to the formation of a fibrin and globulin meshwork, and remain visible on NCT for approximately 1 week (58, 59).

Overall, NCT is inferior to MRI for detection of ischemic infarction (15, 30). Infarct detection with NCT in the first 3 h following a stroke has poor sensitivity, as low as 25% (30, 60). Another major drawback of NCT is that it provides solely structural rather than physiologic information. It cannot reliably differentiate between penumbral and irreversibly damaged tissue (30). Nonetheless, there are three main stages (acute, subacute, and chronic) used to describe NCT manifestations in ischemic strokes (34).

In the acute stage (<24 h), cytotoxic edema changes are subtle on NCT and include loss of normal gray matter to white matter interface and effacement of cortical sulci, which may result in “loss of the insular ribbon sign” or in the “disappearing basal nuclei sign,” which are established changes observed in middle cerebral artery strokes in people. These findings occur due to partial disappearance or loss of definition of the gray-white matter interface (30, 61). A thrombus in the proximal middle cerebral artery may also be observed in the acute phase as an area of hyper-attenuation (30, 34).

In the subacute stage (24 h to 5 days), vasogenic edema leads to greater mass effect, hypoattenuation, and well-defined margins (34).

In the chronic stage (>5 days to weeks), loss of brain tissue and hypoattenuation are observable on NCT (34). In these tissues, hypo-attenuation is highly specific for tissue infarct (30, 62) In addition, its extent is predictive for the risk of hemorrhagic transformation, clinical outcome, and final infarct volume (30, 63).

NCT studies for strokes in veterinary patients are subject to the same benefits and drawbacks as in people (64, 65). The lesions observed on NCT with stroke can be either hyper-attenuating or hypo-attenuating, depending on whether they are hemorrhagic or ischemic, with varying degrees and distribution of contrast enhancement and distinction from surrounding tissue. Furthermore, hemorrhagic lesions can be associated with mass effect. Since numerous other etiologies can cause similar imaging findings on NCT, and the more specific yet subtle findings are not always present or appreciable, interpretation of NCT findings should be interpreted with caution when considering potential vascular accidents (30, 64).

## MRI: Conventional T1- and T2-Weighted Pre- and Post-Contrast Imaging

### For Ischemic Strokes (Figure 3)

The classic appearance of an ischemic stroke is a sharply delineated lesion primarily within the gray matter that is hypointense to surrounding tissue on T1-w, hyperintense on T2-w and fluid attenuated inversion recovery (FLAIR), with weak to no contrast enhancement in the periphery 7–10 days after the onset of stroke (4, 7, 29, 66, 67). Cerebral ischemia becomes visible on T2-w images 6–12 h after the onset of signs, but is usually not seen until at least 8 h after the ischemic insult (68, 69). Furthermore, changes consistent with cerebral ischemia are evident earlier on T2-w FLAIR images than conventional T2-w images (68).

**Figure 3:**
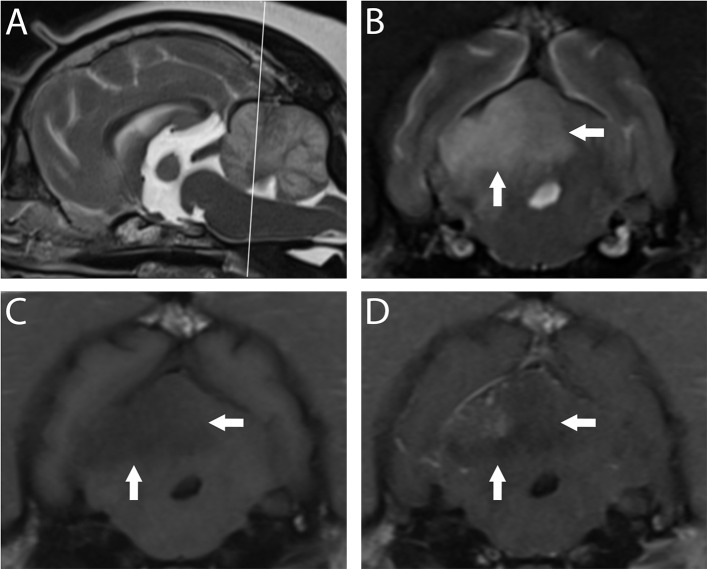
3T MR of the brain of a 10-year-old spayed female Pug with acute right-sided vestibular syndrome of estimated 6–16 h duration at time of imaging. In T2-w sagittal **(A)**, the reference line indicates the orientation of the transverse planes. In T2-w **(B)** the territory of the right rostral cerebellar artery (white arrows) is hyperintense and swelling causes mild leftward midline shift. This region is hypointense in T1-w **(C)** with patchy contrast enhancement of the cerebellar cortex and meninges **(D)**. Findings are consistent with ischemic stroke; however, this combination of signals suggests a lesion several days older than the reported history. The dog is facing to the left in A, and the patient's right is shown on the left in transverse images; this image orientation is maintained throughout this review.

The overall sensitivity and specificity of conventional MRI protocols that include T2-w, T2-w FLAIR, and post-contrast images for ischemic strokes shortly after injury in dogs is poor (70–72). Substantial overlap in signal characteristics and lesion morphology between vastly different intracranial pathologies in dogs has been observed (70–72). In one study using conventional, high-field MRI to compare gliomas and cerebral infarcts in dogs, as many as 12% of histologically confirmed gliomas were incorrectly classified as infarcts (71)/ Additionally, the sensitivity for histologically-confirmed vascular events was 38.9% (70). Furthermore, as many as 47% of presumed cerebrovascular accidents were misdiagnosed as gliomas by reviewers who retrospectively reviewed MR images without knowledge of basic case information (70). Gliomas are characterized as round or ovoid enhancing lesions surrounded by some degree of vasogenic edema, which are features that are generally not encountered in the initial hours following stroke onset (73) Ultimately, the delay between canine stroke onset and imaging contributes to the challenge in distinguishing strokes from gliomas. Additional sequences (see below in Diffusion Weighted Imaging section) have been demonstrated to aid in the distinction.

### For Hemorrhagic Strokes (Figure 4)

In the case of hemorrhagic strokes, T1- and T2-w sequences are fairly sensitive for blood detection, but lack specificity (72). Hemorrhagic strokes can be confirmed on MR imaging because blood breakdown products are paramagnetic (74).

**Figure 4:**
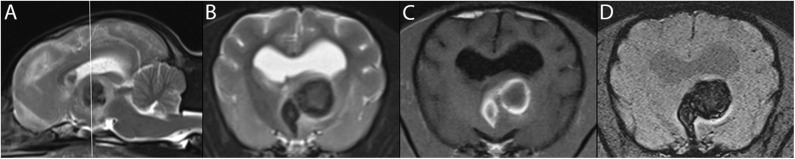
3T MR of the brain of an 11-year-old female American Hairless Terrier, 3 days after acute onset of mentation changes and compulsive circling to the left. The reference line in T2-w sagittal **(A)** indicates the orientation of the transverse planes. In T1-w **(B)**, a large hemorrhagic stroke is present in the left thalamus. The hyperintense ring in T1-w matches a hypointense ring in T2-w **(C)**, consistent with early subacute stage of 3 days or more. The center of the lesion is of intermediate signal intensity in these same sequences, suggesting an earlier stage of hemorrhage. GRE sequences including SWI **(D)** demonstrate signal drop out, confirming the presence of blood products. The patient showed steady improvement on progress examination 3 weeks later.

The appearance of intracranial hemorrhage is multifactorial and depends on intrinsic technical and biological variables (57). While the age of the hematoma is considered the main intrinsic contributor to its signal intensities, size of the lesion, intra- or extra-parenchymal location, and episodes of recurrent bleeding will influence MRI findings (7) Higher field strengths result in an increased T1 relaxation time (75). A near linear relationship has been described between field strength and magnetic susceptibility. Higher field strengths result in an increased T1 relaxation time, which reduces contrast between gray and white matter (74).

The transition from hyperacute to acute hemorrhage is characterized by a transition from oxygenated to deoxygenated blood (7). The different stages have different magnetic properties based on whether or not they contain unpaired electrons (7). This process generally starts at the periphery, leading to a rim effect to the lesion (7).

**Figure 5:**
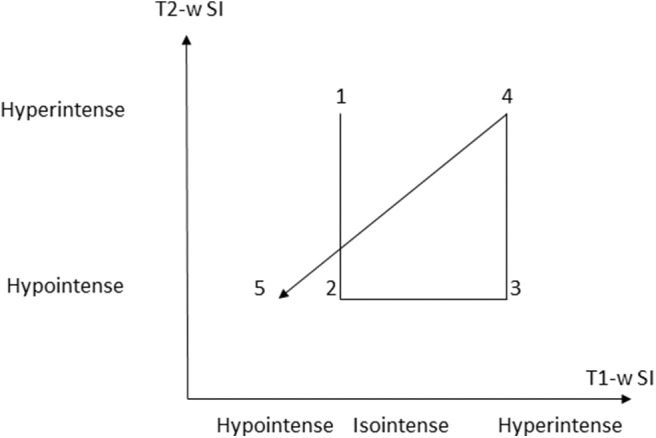
Graphic depiction of appearance of the 5 temporal stages of hemorrhage on T1-w and T2-w sequences. (SI = signal intensity). Modified from Maizlin Z, Shewchuk J, Clement J. Easy ways to remember the progression of MRI signal intensity changes of intracranial hemorrhage. Canadian Assocn of Radiologists Journal 2009; 60:88-90.

Five distinct stages of MRI appearance of intracranial hemorrhage have been defined (7, 57, 76–78) (Table 1) (Figure 5). The hyperacute (<12 h) stage appears isointense on T1 and hyperintense on T2-w images (7, 57, 76–78) The acute (1–3 days) stage is associated with intracellular deoxyhemoglobin, appearing iso- to hypointense on T1-w and hypointense on T2-w (7, 57, 76–78). The early subacute (4–7 days) stage is associated with intracellular methemoglobin with intact red blood cells, appearing hyperintense on T1-w and hypointense on T2-w images (7, 57, 76–78). Methemoglobin produces T1-w shortening effects on adjacent hydrogen nuclei in water and other molecules, leading to intrinsically high signal intensity on T1-w images (7, 57, 76–78). The late subacute (7–14 days to 1 month) is associated with extracellular methemoglobin and erythrolysis, appearing hyperintense on both T1-w and T2-w (7, 57, 76–78). In the chronic (>14 days) stage, ferritin and hemosiderin conversion and storage within macrophages results in iso- to hypointense T1-w and hypointense T2-w lesions (7, 57, 76–78). This final stage may last indefinitely, although recent evidence suggests that it may also resolve with time (79).

**Table 1:** T1-w and T2-w characteristics of hemorrhagic stroke lesions by stages of hemoglobin breakdown.

**Phase**	**Time**	**Compartment**	**Hb product**	**T1-w**	**T2-w**
Hyperacute	<24 h	Intracellular	OxyHb	Isointense	Hyperintense
Acute	1–3 days	Intracellular	DeoxyHb	Isointense	Hypointense
Early subacute	>3 days	Intracellular	MetHb	Hyperintense	Hypointense
Late subacute	>7 days	Extracellular	MetHb	Hyperintense	Hyperintense
Chronic	>14 days	Extracellular	Hemosiderin	Hypointense	Hypointense

*Five distinct stages occur based on the hemoglobin (Hb) breakdown product. OxyHb, Oxyhemoglobin; DeoxyHb, deoxyhemoglobin; MetHb, methemoglobin*.

Three pulse sequence strategies are routinely employed for evaluation of cerebral hemorrhage and should be simultaneously examined: T1-w, T2-w, and GRE (below). T1-w hyperintensity alone is unspecific and may represent various blood breakdown products, fat, proteinaceous fluid, melanin, calcification, necrosis and other paramagnetic substances such as iron, manganese, and copper (1, 73, 80–86).

### T2-w Fluid Attenuated Inversion Recovery (FLAIR) (Figure 6)

FLAIR, along with several other sequences, was developed to evaluate white matter, including in strokes (87, 88). Canine models have been used to track the appearance of ischemic strokes over time on T2-w FLAIR (88–90). Based on these studies, ischemic stroke generally becomes visible within the first 3–8 h after a stroke (7, 30, 88–93). T2-w FLAIR images are also highly sensitive in detecting any fluid-rich lesions, including subarachnoid hemorrhage and acute cerebral venous sinus thrombosis, which both appear hyperintense on T2-w FLAIR (30, 34, 94–96). T2-w FLAIR is particularly useful for evaluating possible lesions adjacent to areas filled with cerebrospinal fluid or in areas normally filled with cerebrospinal fluid, because FLAIR suppresses the signal intensity of bulk water (97).

**Figure 6:**
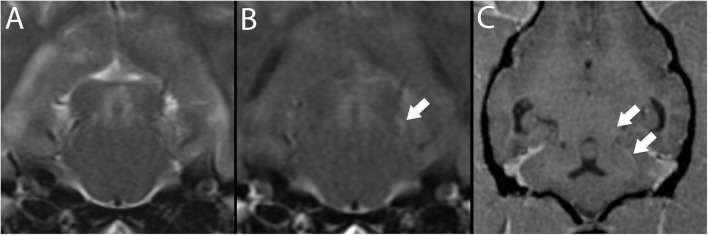
3T brain MR of a 7-year-old neutered male Pug with ischemic stroke of the territory of the left rostral cerebellar artery (not shown). Depicted are T2-w transverse plane at the level of the caudal colliculus **(A)** and matching slice in T2-w FLAIR **(B)**. Depicted in B, a hyperintense vessel sign is present (white arrow). This vessel, consistent with the base of the rostral cerebellar artery, may be followed in T1-w post-contrast MPR in dorsal reconstruction arrows, **(C)**.

Inter-observer agreement on T2-w FLAIR sequences of ischemic lesions is considered excellent (69). T2-w FLAIR specificity and positive predictive value are high for ischemic lesions <5 h old, but sensitivity and negative predictive value are low (92, 98–100). Therefore, in people, a substantial number of patients in this hyperacute time window who could benefit from thrombolytics are missed if solely evaluated based on T2-w FLAIR, as the intravenous use of this drug is only effective if given within 4.5 h of stroke onset (98).

Strokes in many dogs are diagnosed in the subacute stage, between 24 h to 6 weeks following vascular insult (9, 16, 88, 101). This delay relative to diagnosis in people is due to time lag between onset of signs and referral for diagnostic imaging as well as lack of standardized imaging protocols for strokes in dogs (9, 16, 88, 101). In dogs, the median interval between onset of signs and MRI exceeds 2 days, which explains why reported lesions have been hyperintense on T2-w FLAIR (9, 16, 88, 101). Over time, the sensitivity of T2-w FLAIR increases, which can help in identifying strokes even if the interventional window has long passed (90). Although reaching a diagnosis of an ischemic stroke within the critical time window of 4.5 h for administration of thrombolytics is an unlikely expectation in dogs, these studies have provided insight regarding the appearance of strokes over time, which can aid in demarcating lesion size and predicting recovery time (89, 90).

T2-w FLAIR can be used to evaluate two findings consistent with strokes <24 h old: hyperintense vessel signs and hyperintense, swollen cortical gyri (97, 102). In some cases, a hyperintense vessel sign can be the only indication of infarction (97, 102). This sign has been established to result from slow flow, in both anterograde and retrograde leptomeningeal directions, which may explain why its detection is substantially reduced in post-contrast T2-w FLAIR images (103–105).

The sensitivity of these findings is highest during the first 6 h after stroke onset and declines over time (96, 105). In people, the most common locations for this finding on T2-w FLAIR are the sylvian fissure (87%), cortical sulci (54%) and horizontal segments of the middle cerebral arteries (97, 106). Furthermore, infarcts with a signal intensity ratio of <1.37 on FLAIR are <36 h old (68).

Additionally, in people, delayed post-contrast T1-w FLAIR images can be used to detect the presence of the hyperintense acute reperfusion marker (HARM) sign, which is potentially an indicator of early blood-brain barrier disruption (53). In evaluating chronic ischemic infarcts, lesion outlining on FLAIR compared to T2-w images leads to superior inter-rater agreement for lesion borders because it causes a more distinct border between tissue and cerebrospinal fluid compared to T2-w images (107).

### Susceptibility-Weighted Imaging/Gradient Echo (Figures 4, 7)

Combining susceptibility-weighted imaging (SWI) or gradient echo (GRE) imaging such as T2-^*^ with conventional T1-w and T2-w images can increase both the sensitivity and specificity for hemorrhagic strokes (29, 73, 80, 108). GRE MRI uses a gradient to rephase protons, which makes susceptibility effects more visually prominent (7, 109). Hemorrhage appears an-intense (signal void) lesions on these sequences, as hemosiderin is strongly paramagnetic (7, 74, 109–113). Although several other substances can create hypointense lesions on T2^*^-GRE and SWI, such as mineralization, gas, fibrous tissue, and iron deposits, these alternative findings (aside from iron deposits) are both T1-w and T2-w hypointense, unlike hemorrhagic stroke lesions (45, 74). On T2^*^-w images, chronic hemorrhage is characterized by a hyperintense center surrounded by a hypointense rim (114).

**Figure 7:**
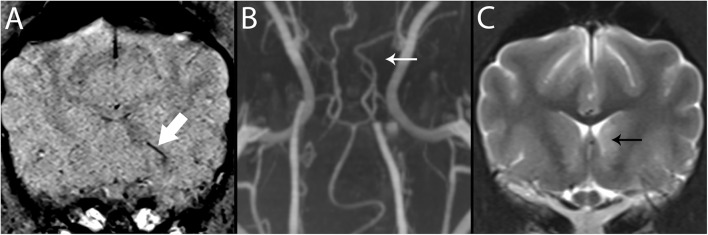
3T MR of the brain of an 8-year-old neutered male Greyhound with ischemic stroke to the left caudate nucleus. In SWI **(A)**, Susceptibility Vessel Sign is present (bold arrow). In TOF **(B)**, a branch of the rostral cerebral artery is not identified (arrow), corresponding to the vessel shown in A, and to the recurrent artery of Heubner which feeds the basal nuclei in this region. In T2-w **(C)**, immediately caudal to the level shown in A, the left caudate nucleus is hyperintense (black arrow).

Compared to CT, GRE is equally, if not more, sensitive for detection of acute intracranial hemorrhage (115–117). Currently, T2^*^-GRE is the most commonly used sequence for evaluating hemorrhage in dogs and has been demonstrated to be the most accurate of all MR pulse sequences and more accurate than CT in dog models in predicting the extent of hemorrhage (23, 80). T2^*^-GRE sequences are not 100% sensitive for the detection of all stages of intracranial hemorrhage because after erythrolysis, methemoglobin moves into the extracellular space and becomes homogeneously distributed in plasma (73) This means that the magnetic field within the voxel also becomes homogeneous, which causes loss of susceptibility artifact (73, 118). Furthermore, the artifact distortion is directly proportional to the magnetic field strength, so the size of hemorrhage can vary between scanners (23, 73). Subsequently, SWI has been demonstrated to be more reliable for cerebral microbleeds than T2^*^-GRE (98).

### Diffusion-Weighted Imaging (Figure 8)

Diffusion-weighted imaging (DWI) provides an image signal that is dependent on the molecular motion of water (30, 35). The disruption in energy metabolism that results from ischemic stroke leads to failure of the sodium-potassium pump, resulting in cytotoxic edema (30, 119). Intracellular water flow leads to reduced extracellular volume. Within the extracellular space, water mobility is more facilitated than within the intracellular space. Given the altered distribution of water between these two compartments, a net reduction of Brownian motion and osmosis results (30, 35, 120). This diffusion impairment is detected on DWI within minutes of vessel occlusion as a well-demarcated hyperintense signal on DW source images (30, 34, 35, 120). The defined area tends to be larger than the area of tissue that directly experiences ion pump failure, amplifying its visibility (67, 121). Additional pathologic processes can contribute to the DW source images' hyperintensity, including alterations in pH at the periphery due to anaerobic metabolism (67, 121).

**Figure 8:**
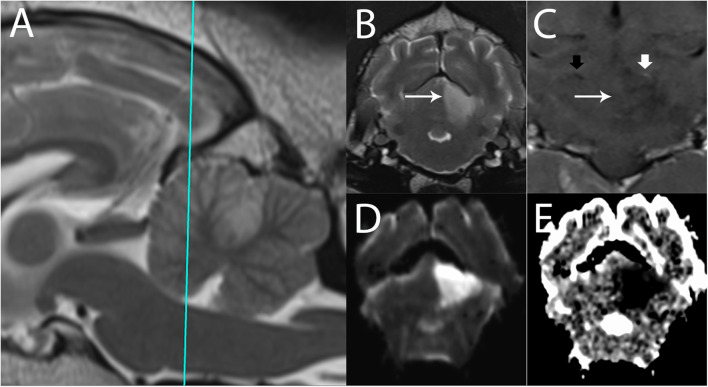
3T MR of an 11-year-old spayed female boxer dog with ischemic stroke of the left rostral cerebellar artery, 2 days after onset of signs. The patient presented mentally appropriate in lateral recumbency with opisthotonus and decerebellate rigidity. Shown are **(A)** T2-w sagittal with a reference line for the transverse planes **(B)** T2-w transverse, **(C)** T1-w FLAIR dorsal after administration of contrast **(D)** Trace DW, and **(E)** ADC map in transverse plane. In the left rostral cerebellum, there is a well-defined, wedge-shaped T2-w hyperintense area (B, arrow). This lesion is hyperintense in Trace DW **(D)** and hypointense in ADC map, consistent with restricted diffusion. In dorsal plane **(C)**, the edge of the lesion is marked by the long arrow. Intravascular enhancement of the left rostral cerebellar artery is present (thick white arrow, compare with the right, thick black arrow); vascular enhancement is a common finding and is related to slow flow. No predisposing causes were found on bloodwork and abdominal ultrasound. The patient gradually improved over 14 days and was subsequently discharged as ambulatory with mild cerebellovestibular ataxia.

An apparent diffusion coefficient (ADC) map is constructed to quantify the extent of restricted diffusion observed on the DWI image (30). ADC maps quantify the degree of water proton mobility between magnetic fields. Normal ADC values vary substantially depending on the brain region and the age of the dog (122, 123). True reduced diffusion, as occurs in cytotoxic edema, appears hypointense on the ADC map, confirming acute ischemia (7, 29, 72, 124, 125). The comparison between the DWI and ADC is necessary because DWI hyperintensity is not exclusively specific for restricted diffusion. Additional conditions that lead to restricted diffusion include pyogenic abscesses, highly cellular tumors, status epilepticus, and global ischemia (126). Areas of high signal, such as vasogenic edema, can also appear hyperintense on DWI because DWI is T2-w-based (7, 9, 34, 125). Vasogenic edema, being extracellular edema, will not show restricted diffusion and will appear hyperintense on an ADC map, by a phenomenon termed T2-w-shine through (7, 9, 34, 123). Over time, the appearance of the DWI and ADC abnormalities reverse as the stroke moves into a subacute phase within 24 h to 5 days (34, 35). This progression makes it possible to estimate the age of the infarct core to some degree (34, 35, 88).

DWI is most sensitive for detecting ischemic strokes immediately following onset. Changes can be observed as early as 45 min following a stroke (127). In addition to T2-w FLAIR, it is the most sensitive method for detecting ischemic infarcts with high diagnostic accuracy and is considered the gold standard for identifying acute stroke in people (115). Once a hemorrhagic stroke has been ruled out, DWI improves stroke detection from 50% to more than 95% (34, 40, 128). On DWI, the sensitivity and specificity in diagnosing hyperacute cerebral infarction is 88–100% and 86–100%, respectively (126). DWI is also more sensitive for smaller lesions than CT and more accurately reflects pathophysiologic changes induced by acute ischemia compared to T2-w images and is also a better predictor of final infarct volume (30, 127, 129). Another advantage of DWI is its capability to discern between acute vs. chronic ischemia, which is useful in identifying new lesions in patients with prior ischemic injury (30). It also is useful in distinguishing between ischemic stroke and glioma. As the ADC value is inversely proportional to cell density, high grade gliomas generally have a classically lower ADC value in comparison to ischemic tissue (73).

**Figure 9:**
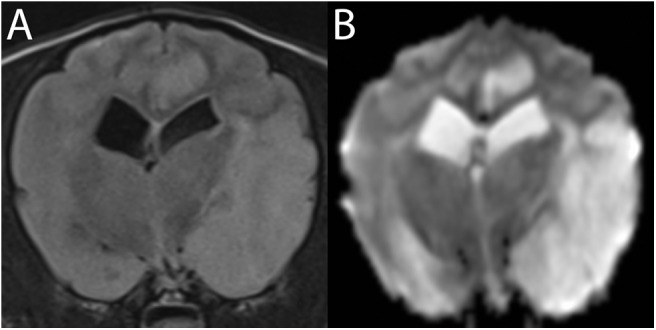
3T MR of the brain of an 8-year-old neutered male Cavalier King Charles Spaniel with severe endocardiosis. In T2-w FLAIR **(A)** the territory of the left middle cerebral artery is moderately hyperintense and swollen. The same lesions are much more conspicuous in Trace DW, with the hyperintensity being consistent with edema **(B)**. Similar lesions in the left cingulate gyrus are consistent with ischemic stroke of a branch of the rostral cerebral artery. Following the patient's cardiovascular arrest 2 days later, necropsy confirmed acute to subacute ischemic stroke.

Comparing findings between FLAIR and DWI can help determine the age of an ischemic stroke (Figure 9). In patients evaluated shortly after development of signs, a DWI lesion and normal FLAIR image suggests a time window of <3 h with >90% specificity and positive predictive value for an ischemic stroke (30, 89, 92, 100). This mismatch is critically important in human medicine in identifying candidates for thrombolysis, which is only effective if delivered within 4.5 h of injury (30, 89, 92, 98, 130). Furthermore, use of a calculated DWI-FLAIR mismatch increases sensitivity over visual mismatch analysis (89).

There are several limitations and important considerations regarding DWI and ADC imaging. For one, DWI lesions can be at least partially reversible in the early stages of ischemia. The initial DWI abnormality therefore does not necessarily reflect the infarct core (30, 131). Additionally, in the time frame in which DWI is most sensitive for detecting strokes, the greatest variability in lesion measurement between observers has been documented (132). Even with manual editing and the development of threshold values, ADC can include visibly normal tissue and miss visibly abnormal tissue (133). Furthermore, changes on ADC 3 h after an ischemic event are not reliable predictors of the reversibility of tissue damage (89).

Currently, DWI and comparison to the ADC map is the most widely used and most sensitive and specific sequence used in evaluating strokes in dogs (7, 9, 64). In a study of 40 dogs with suspected infarcts that underwent MRI within 1–5 days of presumed stroke onset, lesions were more readily visible on DWI than on conventional fast spin echo images (16).

## Perfusion Studies

### MR Perfusion-Weighted Imaging (MR-PWI)

Comparison of DWI images with perfusion-weighted imaging (PWI) images can be useful in determining the fate of ischemic tissue. PWI provides a measurement of cerebral perfusion by tracking the passage of an intravenously delivered bolus of contrast agent (115, 134). The temporal passage of contrast is tracked in repeated contiguous slices throughout the brain using gradient echo techniques (115). The tissue signal change caused by the susceptibility effect of contrast is used to create a hemodynamic time-to-signal intensity curve (115, 135). This curve is then used to generate a set of semiquantitative perfusion maps (112, 132). The maps establish relative cerebrovascular hemodynamic measures such as relative CBF, MTT, CBV and time-to-peak (TTP) (115, 129, 130, 136, 137). Ischemic tissue has increased MTT, decreased CBF, and normal CBV (61). Infarcted brain tissue, or necrosis due to complete and prolonged ischemia, has increased MTT, decreased CBF, and markedly decreased to no CBV (61).

One of the major uses of PWI in stroke patients is to identify at-risk yet salvageable penumbra tissue by detecting perfusion-diffusion mismatches when comparing lesions on DWI to PWI (61, 138–141). DWI detected abnormality reflects the irreversibly damaged infarcted core, whereas PWI reflects the overall area of hypoperfusion (61). Mismatch is defined as PWI lesion volume that exceeds the DWI lesion volume by at least 20% (137).

There are substantial drawbacks to evaluating the PWI/DWI mismatch. The model does not take into consideration that DWI lesions do not necessarily turn into infarction and that PWI abnormalities might represent areas of benign oligemia that are not at risk (115, 132, 141). Despite reported correlation of the PWI/DWI mismatch with salvageable vs. irreversible tissue damage, there are reports of salvageable tissue that was hyperintense on initial DWI (67, 142, 143). Therefore, the reliability of this comparison might not be as strong as previously considered (67).

Although PWI is not performed conventionally in veterinary medical practice, numerous studies using canine models have been performed (88). Therefore, PWI could be employed in MRI protocols in canine patients with suspected strokes. However, as currently there are no specific therapies aimed at restoring penumbral or oligemic tissue, its application may be impractical and could prolong time under general anesthesia. Another limitation of PWI in veterinary medicine is the highly equipment-dependent variation in resolution and/or strong distortion artifact.

### CT Perfusion Imaging

Perfusion imaging is also feasible using CT and offers several advantages over non-contrast CT and over MR-PWI. CT perfusion imaging is performed using a single injected bolus of iodinated contrast material. The passage of the bolus is tracked through the cerebral circulation under sequential helical CT scanning. Similar to MRI-PWI, CBF, CBV, TTP, and MTT are all acquired using CT perfusion imaging, and the same patterns attributable to infarcted and penumbral tissue are visible on CT perfusion. Both CT perfusion imaging and MR-PWI may derive CBV, CBF, and MTT, among others, as quantitative data (144). The main advantages of CT perfusion imaging over NCT is the ability to use hemodynamic differences to evaluate intracranial vascular physiology. The main advantage of CT perfusion imaging over MRI-PWI is the shorter time to image acquisition with CT perfusion imaging (145).

## Vascular Studies

### Computed Tomography Angiography (CTA)

This is the most common first-line diagnostic modality for vascular imaging in acute strokes in people (146, 147). This minimally invasive study requires a time-optimized bolus injection of intravenous contrast material and thin-section helical CT images obtained in the arterial phase (132, 146, 148, 149). CTA can reliably detect intracranial proximal arterial occlusions and stenosis (146, 150). The presence of occlusion on CTA predicts functional outcome, final infarct size, and response to intravenous thrombolytics (146, 149, 151). It can also provide information on the quality of collateral circulation if the scanner possesses sufficient spatial resolution capability and can improve the sensitivity in identifying ischemic areas that are not apparent on non-enhanced CT (30, 146, 152–155). Post-processing techniques can also be used to create three-dimensional images that can aid in detection of arterial occlusions (146, 155, 156). CTA is widely available and well-tolerated by the majority of patients, and provides crucial information in a short period of time (34, 146).

Although CTA is the established modality for evaluating strokes in people, there are no published studies on using CTA in dogs. This paucity is likely due to several reasons. For one, most dogs undergo imaging long after the possible administration of thrombolytic agents would have any benefit. The main reason that CTA is the most common modality chosen for human stroke patients is its rapidity and high sensitivity for arterial occlusion and hemorrhagic infarcts. This means that it can provide the fastest way to reach a diagnosis and provide an opportunity for administration of thrombolytic agents within a 4-h window of stroke onset. Additionally, the clinical signs of stroke in dogs overlap with other etiologies, such as brain tumors and immune-mediated meningoencephalitis. Whereas, strokes are one of the most common neurologic diseases in people and their signs are more apparent to the general public, the same principles do not apply to strokes in dogs. Therefore, MRI is preferred over CT, and CTA is not often pursued because the standard MRI protocol for dogs generally yields a definitive diagnosis for stroke patients. Furthermore, in most cases, general anesthesia is required for CT angiographies in dogs, so the reduction in time spent obtaining a diagnosis via CT compared to MRI is relatively minimal.

### Magnetic Resonance Angiography (MRA) (Figures 7, 10)

Like CTA, MRA is used to visualize both intracranial and extracranial vasculature (30). The two most commonly used types of MRA are time-of-flight (TOF-MRA) and contrast-enhanced MRA (CE-MRA) (146, 157). Both are based on gradient echocardiographic sequences with either two-dimensional or three-dimensional volume acquisition (17).

**Figure 10:**
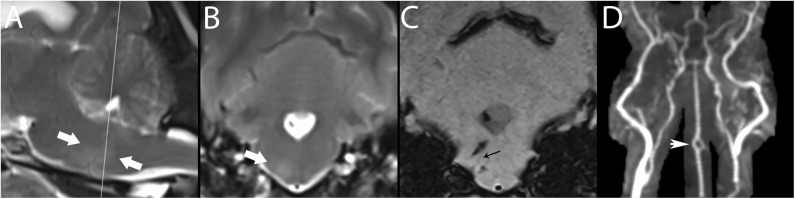
3T MR of the brain of a 7-year-old female spayed Maltese with central vestibular disease secondary to hemorrhagic stroke. Centered on a right-sided perforating pontine vessel, there is a T2-w hyperintense halo, shown here between arrows in paramedian **(A)** and transverse **(B)** planes. In SWI **(C)**, linear signal void is associated with the vessel (black arrow); linear hemorrhagic lesions are present dorsal and ventral to this vessel, as well as in the fourth ventricle. While pontine vessels are not clearly visualized in TOF **(D)**, large artery disease is ruled out, classifying this lesion as basilar artery branch disease. CE-MRA may resolve such small vessels. The basilar artery is fenestrated, thought to be an incidental finding (white arrowhead). Necropsy confirmed the stroke and identified an adrenal pheochromocytoma, presumed to have resulted in systemic hypertension, a major risk factor for stroke.

Three-dimensional (3D) TOF-MRA is the standard technique for examination of intracranial vessels, although CE-MRA is also recommended in protocols for ischemic brain disease (93, 115, 158). TOF-MRA does not require a contrast agent and is instead based on macroscopic motion of water proteins (157, 159, 160). In this sequence, saturation pulses are repeatedly applied to a volume of tissue; inflowing blood is unsaturated and provides signal against a background of low intensity (93, 115, 158). TOF-MRA is ideal for evaluating arterial flow because venous blood is suppressed except on a few entry slices due to its slower flow and thicker slab (161). Additionally, TOF-MRA can be used to evaluate venous blood by applying a saturation band and pulse on the opposite side (162).

Limitations of TOF-MRA are based mainly on vascular saturation and flow or motion artifacts. In 3D-TOF-MRA, volume coverage is limited by vascular saturation effects, making it best suited for evaluation of intracranial rather than extracranial vasculature (146, 150, 163, 164). 3D-TOF acquisitions have intrinsically higher spatial and contrast resolution, a characteristic which is amplified at higher magnetic fields, so the ability to follow vessels is superior in 7.0T scanners compared to 3.0T scanners (7, 17) TOF-MRA also tends to overestimate the degree and length of arterial stenosis, especially in the presence of turbulence, is relatively insensitive to slow or reversed blood flow and tends to be affected by saturation effects that limit the maximum thickness of slabs acquired (141). This factor means that it can be difficult to visualize the distal aspect of vessels (146). Furthermore, slow flow or large blood volume can eliminate the visible intrinsic contrast between blood and stationary tissue (165).

CE-MRA is the standard technique for examination of extracranial arteries such as the vertebral or carotid arteries (30, 146). It utilizes an intravenous injection of gadolinium to reduce the T1-w relaxation time of tissue and to generate contrast between the intravascular lumen and surrounding tissues (17, 30, 135, 146, 164). Since it is independent of flow dynamics, unlike TOF, artifacts or effects associated with altered flow are substantially reduced (30, 146, 164).

Unlike CTA, MRA has been studied relatively extensively in dogs. Prior to its availability, and given lack of CTA studies, the intracranial vessels of dogs were historically evaluated using conventional x-ray angiography or digital subtraction angiography, which require iodinated contrast agents and are relatively invasive techniques (17, 41, 165). MRA has now been used in numerous studies of both normal anatomic structures and pathologic processes in dogs, including strokes (7, 17, 41, 157, 165–170). In assessing canine strokes, it has been demonstrated to be a relatively easy sequence to include in an MRI protocol when a stroke is suspected (17).

CE-MRA has been demonstrated to show all major intra- and extra-cranial arteries and veins and the venous sinuses and plexuses of the canine brain (151). Its use may be limited by magnetic field strength; 1.5T units may not be sufficient for producing images that can be interpreted easily, and while 3T units provide the same image quality as 7T units, quantitatively, more vessels may be visible on 7T units (17). A study performed using a 1.5T unit facilitated visualization of the rostral cerebral artery, cranial and caudal communicating arteries, middle cerebral artery, and the rostral cerebellar artery (157). There is discrepancy regarding whether or not the rostral cerebellar artery is readily visible on scanners other than 7T units. In one study, the rostral cerebellar artery was not reliably observed (17). In another study that used a 1.5T scanner, the rostral cerebellar artery was the only cerebellar artery that could be reliably detected (157).

TOF-MRA has demonstrated efficacy in confirming vessel occlusion in a canine experimental model of permanent occlusion of the middle cerebral artery (157). When evaluated 2–3 days following occlusion, the angiograms of all dogs demonstrated either complete or partial flow attenuation, depending on the degree of vessel occlusion (157). Overall, based on the relative ease of performing MRA and the diagnostic utility, either TOF-MRA or CE-MRA, or ideally both, should be incorporated into canine MRI protocols when an ischemic stroke is suspected. One logistical benefit of CE-MRA over TOF-MRA is the time to image acquisition. CE-MRA, typically performed as T1-w fast-field echo (T1-FFE) or fast low-angle shot (FLASH) sequences, have scan times in the 10–30 second time range and allow for breath-hold acquisitions. TOF-MRA scan times range from 8–10 min for human heads, with similar ranges expected for dogs (171).

## Section 3: Select Emerging Sequences

### Measuring Cerebral Blood Flow

PWI has been demonstrated to provide useful information regarding hemodynamic status in human patients actively undergoing stroke or at risk for stroke (30, 115). However, its use in human medicine is limited clinically by the risk of contrast-associated nephrogenic systemic fibrosis in patients with moderate to severe renal impairment (115, 172). This has led to the development of new studies of cerebral blood flow and hemodynamics that do not require gadolinium-based contrast (115). These sequences are newly emerging in human medicine and have yet to be investigated in veterinary patients.

### Arterial Spin Labeling MRI

Currently, arterial spin labeling (ASL) is the most popular MRI hemodynamic analysis method that does not require exogenous contrast administration (115). To perform ASL, radiofrequency pulses are applied to the blood water proximal to the tissue of interest (115). A delay time is permitted and the radiofrequency-labeled blood water protons travel to the brain and exchange with tissue water (115). This leads to a small but measurable reduction in the tissue water magnetization proportional to the amount of exchange, which approximates CBF (115). The change is relatively small compared to the total amount of signal, so it is amplified by subtracting the images from a control image (115). Models of tracer kinetics are then applied to the ASL difference signal to quantify cerebral blood flow in units of milliliter of blood per 100 grams of tissue per minute (115, 173).

There are numerous ASL techniques that differ regarding the labeling application. In general, they can be grouped into two categories- pulsed ASL and continuous ASL (115, 174–176). Pulsed ASL uses one or two radiofrequency pulses of a duration of 3–15 milliseconds, which labels blood water over a large volume (80–120 millimeters) in the neck (115, 175, 176). Continuous ASL uses a long labeling pulse (1.5–2 s) at a single location in the neck (115, 177, 178). The signal to noise ratio in continuous ASL is 30–50% higher than pulsed ASL, so in principle continuous ASL is more desirable (115, 179). However, it requires special local transmit coils, whereas pulsed ASL can be performed using standard MRI coils (115, 180). A new labeling strategy is vessel-selective ASL (115, 181–183). In this technique, different feeding arteries, such as the left and right internal carotid arteries and basilar artery are separately labeled (115, 181–183). This gives a measure of perfusion territories and collateralization (112, 181–183).

There are currently no canine studies evaluating the use of ASL. ASL is a useful technique in human stroke evaluation because it does not require administration of a contrast agent. This eliminates the risk of contrast administration-associated morbidity. This appealing feature could be exploited in diagnostic investigation in dogs with suspected strokes and its use may reduce scan time. Based on clinical suspicion of a stroke, could be performed as one of the first sequences, eliminating the need for subsequent time-consuming, low-yield sequences.

## Tractography

### Diffusion Tensor Imaging (DTI) (Figure 11)

DTI-based tractography is a technique used to localize specific neuronal white matter fiber tracts (73). It is used in evaluation of intracranial space-occupying lesions such as brain tumors and vascular malformations associated with white matter (73, 184–189). It has also been used in stroke imaging to assess the relationship between fiber tracts and infarcts (73, 115, 190, 191). DTI was developed to visualize the orientation and properties of white matter and relies on anisotropy, or the direction-dependent diffusion of water (73). Diffusion is faster in the direction of fiber tracts rather than perpendicular to them (73). This is represented mathematically and graphically by a diffusion ellipsoid or tensor (73). When there is no directionality, the tensor is spherical, whereas in white matter tracts, the tensor is normally cigar shaped (73). The tensors of cerebral white matter can then be reconstructed to track the three-dimensional orientation of the fibers. The DTI data are also presented as two-dimensional fractional anisotropy maps. (73) Fractional anisotropy has been shown to decrease after stroke disintegration of gray-white matter distinction (73, 190, 191). It has demonstrated a fair correlation with clinical signs and may also be used to predict human patient outcomes after stroke (73, 192, 193). The main drawback to DTI-based tract-graphs is that it is not fully validated (73, 194–198). Additionally, tractography may underestimate or overestimate the quantity of fiber tracts (73, 199).

**Figure 11:**
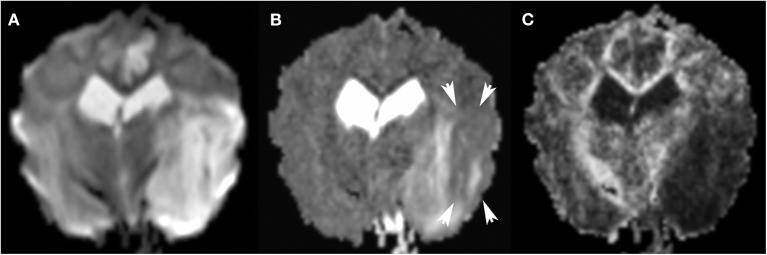
3T MR, same dog as in Figure 9. The large areas of hyperintensity in Trace DW **(A)** are greatly attenuated in ADC map **(B)**: persistent hyperintensity corresponds to vasogenic edema, and zones which transitioned to ADC hypointensity (arrows) correspond to cytotoxic edema. Many factors may affect the degree of ADC hypointensity, most importantly time since onset. In FA **(C)**, the matching dark region indicates reduced anisotropy.

There are currently no canine studies evaluating the use of DTI in the context of vascular disease. However, currently in progress are feasibility and mapping studies in dogs (200). Fractional anisotropy has been demonstrated to reflect features that constrain water diffusion in white matter, including myelination and other microstructural factors (201). With optimization of these parameters, DTI may become a more useful tool in diagnosing strokes in dogs (200–202).

## Long Term Monitoring

In general, most dogs recover from strokes with time and supportive care only (7, 23, 72). No specific treatment is available that affects the outcome of either ischemic or hemorrhagic strokes in dogs (7, 23, 71). Rather, treatment focuses on preventing secondary brain damage or complications associated with the underlying disease (7, 23).

There are limited studies evaluating the long-term MRI findings of stroke over time. In general, the lesion volume tends to increase in the 24 h to 8 days following stroke onset. Secondary changes, such as vasogenic edema and leukocyte infiltration, can lead to this increased volume, detectable on FLAIR sequences. In the chronic phase, infarct lesion volume decrements due to consolidation and pseudocystic tissue change and tissue loss (203, 204).

## Conclusions

There are numerous established and novel CT and MRI modalities for evaluating stroke that hold promise for application in veterinary medicine. The main limitations for their use include temporal and resource associated factors. In general, few canine patients that undergo a stroke are evaluated in the same acute time frame as in people. This is likely because the features of strokes in dogs are less familiar than those in people. The temporal patterns observed between some of the imaging modalities used in human medicine may not be applicable to veterinary medicine. Additionally, while an increasing number of MR-equipped veterinary facilities are available, the investment in specialized coils or training for advanced techniques is lacking. Furthermore, no studies exist regarding interventions for canine stroke patients, which makes it challenging to defend advanced imaging modalities that add time under general anesthesia but otherwise have little impact on patient outcome. In cases where a stroke cannot be definitively distinguished as the cause of disease using standard T1-w, T2-w, FLAIR, SWI, and DWI/ADC images, the more novel modalities discussed in this review may be beneficial. Ultimately, the translational impact of veterinary use of these modalities may prove to be their most important application, particularly regarding establishing guidelines for long-term outcome in acute strokes.

## Author Contributions

SP contributed to the study conceptualization. SA contributed to the manuscript composition. KG contributed to manuscript editing and image composition. FW contributed to manuscript editing.

### Conflict of Interest

The authors declare that the research was conducted in the absence of any commercial or financial relationships that could be construed as a potential conflict of interest.
